# Cap-Independent Translational Control of Carcinogenesis

**DOI:** 10.3389/fonc.2016.00128

**Published:** 2016-05-25

**Authors:** Beth Walters, Sunnie R. Thompson

**Affiliations:** ^1^Department of Microbiology, University of Alabama at Birmingham, Birmingham, AL, USA

**Keywords:** IRES, cap-independent, tumorigenesis, p53, Apaf-1, c-Jun, apoptosis, translation

## Abstract

Translational regulation has been shown to play an important role in cancer and tumor progression. Despite this fact, the role of translational control in cancer is an understudied and under appreciated field, most likely due to the technological hurdles and paucity of methods available to establish that changes in protein levels are due to translational regulation. Tumors are subjected to many adverse stress conditions such as hypoxia or starvation. Under stress conditions, translation is globally downregulated through several different pathways in order to conserve energy and nutrients. Many of the proteins that are synthesized during stress in order to cope with the stress use a non-canonical or cap-independent mechanism of initiation. Tumor cells have utilized these alternative mechanisms of translation initiation to promote survival during tumor progression. This review will specifically discuss the role of cap-independent translation initiation, which relies on an internal ribosome entry site (IRES) to recruit the ribosomal subunits internally to the messenger RNA. We will provide an overview of the role of IRES-mediated translation in cancer by discussing the types of genes that use IRESs and the conditions under which these mechanisms of initiation are used. We will specifically focus on three well-studied examples: Apaf-1, p53, and c-Jun, where IRES-mediated translation has been demonstrated to play an important role in tumorigenesis or tumor progression.

## Introduction

Gene expression is regulated at multiple levels: DNA, transcription, translation, messenger RNA (mRNA) turnover, mRNA or protein localization, and protein stability. Dysregulation of any one of these steps results in aberrant gene expression, which can be detrimental to the cell or organism. The sheer complexity of these processes and the energy that is invested in them demonstrates the importance of accurate regulation of gene expression. The major research focus in aberrant gene expression in cancer has been on alterations in DNA or transcription. However, the improved methods for proteomics and transcriptomics have revealed that there is a remarkably low correlation between mRNA transcript and protein levels (1), suggesting that protein expression is extensively regulated at the posttranscriptional or posttranslational level. This review focuses on translational control mechanisms during cellular stress. Global mechanisms of translational control in cancer were reviewed elsewhere (2).

The vast majority (>90%) of mRNAs are translated using a cap-dependent mechanism of initiation. Briefly, the eukaryotic initiation factor 4F (eIF4F) complex consisting of eIF4E, eIF4A, eIF4G recognizes and binds to the 5′ cap structure on the mRNA. The 40S subunit is recruited to the 5′ end of the mRNA as a 43S complex (40S, eIF5, eIF3, eIF1, eIF1A, eIF2, and Met-tRNA_i_). The 40S subunit scans down the mRNA in a 5′–3′ direction until a start codon is recognized, the 60S subunit joins, forming an 80S ribosome, and protein synthesis begins (3). Translation is a very energy-demanding process (4); therefore, under conditions of cellular stress, cap-dependent translation is globally downregulated by several mechanisms, depending on the type and extent of the stress. Protein synthesis can be inhibited globally by phosphorylating eIF2α, which delivers the initiator met-tRNA_i_ for each round of initiation (5). There are four kinases that sense distinct cellular stresses and phosphorylate eIF2α. This globally downregulates protein synthesis while simultaneously enhances translation of certain mRNAs that encode for proteins involved in cell adaptation to cellular stress. Some IRESs are less sensitive to eIF2α phosphorylation during global inhibition of translation (6–8).

Mammalian target of rapamycin (mTOR) is a conserved protein kinase that regulates a multitude of cellular processes in response to growth factors or nutrients (9, 10). mTOR forms complexes with other proteins to form two distinct complexes, mTORC1 and mTORC2 (11). The mTORC1 complex is best understood and senses both intracellular and extracellular cues: growth factors, stress, oxygen, energy status, and amino acids. Signals that are pro-growth, such as growth factors, and nutrients stimulate mTOR, whereas stresses inhibit mTOR activity. Inhibition of mTOR results in de-repression of eIF4E-binding protein (4E-BP), which sequesters the cap-binding protein [eukaryotic initiation factor 4E (eIF4E)] that is required for cap-dependent initiation (12). 4E-BP inhibits the most predominant mechanism of translation initiation, cap-dependent initiation. Proteins synthesized from mRNAs under these conditions use a non-canonical, cap-*in*dependent mechanism of initiation from an IRES located in the 5′ untranslated region (5′UTR). Initiation of protein synthesis by an IRES involves internal recruitment of the 40S ribosome either upstream or directly at the start codon by an unknown mechanism that does not require a 5′ cap structure, eIF4E cap-binding protein, or a free 5′ end. IRES-containing mRNAs encode for proteins that are involved in cell growth, proliferation, apoptosis, and angiogenesis. There are several examples in the literature that demonstrate that IRES-mediated translation of certain mRNAs is required for tumor growth or vascularization (13–24) or resistance to apoptosis (20, 25, 26).

Viral IRESs were the first IRESs discovered, followed by the identification of cellular IRESs (27–29). IRESs are structurally and functionally diverse from one another and must be identified using a properly controlled functional assay, thus making identification of IRESs difficult. A commonly used functional assay has been the bicistronic reporter, whereby translation of an upstream open reading frame (ORF) is translated by a cap-dependent mechanism and translation of the downstream ORF relies on an IRES in the intergenic region for translation. The bicistronic reporter is generally transfected into cells as DNA, depending on the cellular machinery to transcribe it into RNA, which is transported to the cytoplasm for translation. However, if there are cryptic splice sites or promoters that are introduced into the intergenic region (either in the IRES or instead of the IRES), then this can result in a transcript that encodes for a chimeric protein or a monocistronic message, respectively. Either way translation of the downstream reporter would be cap-dependent. Therefore, it is important to show not only that the bicistronic reporter yields expression of the downstream ORF but also that translation of the downstream ORF is independent of the first ORF and not from a monocistronic mRNA. Another potential false positive is if both ORFs are in the same reading frame, then there can be readthrough of the upstream stop codon, resulting in a chimeric protein that is translated cap-dependently. While readthrough is easy to control for, the others are difficult since this requires proving a negative that monocistronic mRNAs or alternatively spliced mRNAs are not present even at low levels (30). Since most of these artifacts arise from transfecting DNA, the obvious solution is to transfect RNA to bypass cellular splicing and transcription; however, while this works fine for viral IRESs, many cellular IRESs may require a “nuclear experience,” to bind IRES-transacting factors that are important for stabilizing the IRES structure (31, 32). This may be due to the fact that unlike the highly structured viral IRESs, mRNAs that contain cellular IRESs may switch between cap-dependent and cap-independent translation, depending on the cellular conditions. Stable RNA structures would be detrimental for cap-dependent translation, which involves scanning of the 40S subunit from the 5′ end of the mRNA to the start codon. These challenges have led to the false reporting of some IRESs in the literature. This has resulted in a lot of controversies around cellular IRESs and which one have been correctly identified (33, 34). Nevertheless, there are compelling data demonstrating internal initiation of both cellular and viral IRESs (35–37), as well as their role in cancer development and gene expression during growth and development (16, 17, 24, 38–40).

In this review, we will describe the basic stress conditions that are known to upregulate IRES-mediated translation during tumorigenesis. In order to demonstrate the significance of the role that cellular IRESs have in cancer, we have compiled a table of validated, *bona fide* IRES-containing cellular mRNAs with important roles in tumorigenesis (Table 1). In addition, we provide a detailed review of three cellular mRNAs that contain IRESs and discuss how their translational regulation impacts oncogenesis, cancer progression, and survival of the tumor cells.

**Table 1:** **IRES-containing cellular mRNAs with important roles in tumorigenesis**.

Gene	Cryptic promoter^a^	Cryptic splicing^a^	Readthrough	Reference
AML1/Runx1	No	No	No	(41)
Apaf-1 (apoptotic protease-activating factor-1)	No	No	No	(42)
Cat-1 (cationic amino acid transporter)	No	No	No	(43)
c-IAP1 (cellular inhibitor of apoptosis protein 1)	No	No	No	(44)
cyp24a1	No	No	No	(45)
EGR (early growth response)	No	No	No	(46)
EGFR/ERBB1/HER1 (epidermal growth factor receptor)	No	No	ND	(47)
Hox	No	No	No	(48)
Hif1α (hypoxia-inducible factor 1-alpha)	No	No	No	(14)
c-Jun	No	No	No	(49)
c-myc	No	No	No	(50, 51)
l-myc	No	No	No	(52)
n-myc	No	No	No	(40)
p16^INK4a^/CDKN2A	No	No	ND	(53, 54)
p27	Yes	No	No	(55–57)
p53	No	No	No	(58, 59)
p120	ND	No	ND	(17)
SNAT2 (sodium-coupled neutral amino acid transporter)	No	No	No	(60)
c-src	ND	ND	No	(8)
SREBP-1a (sterol-regulatory-element-binding protein 1a)	No	No	No	(61)
VEGF (vascular endothelial growth factor)	No	No	No	(62–64)
XIAP (X-chromosome-linked inhibitor of apoptosis)	No	Yes	No	(21, 65)
Zeb2	No	No	No	(66)

*^a^The presence of cryptic promoter or splicing activity does not exclude an mRNA from having an IRES, rather this means that a dicistronic DNA reporter cannot be used to validate the presence of an IRES or that there is a problem with the particular reporter construct, and it may present artifacts independent of the IRES being tested*.

*ND, not determined*.

## IRES-Mediated Translation During Cellular Stress

### mRNAs Encoding Proteins Involved in Tumorigenesis and Cell Survival Are IRES-Dependent

Translation is globally downregulated under a variety of cellular conditions, such as mitosis, heat shock, cold shock, hypoxia, DNA damage, osmotic shock, starvation, and apoptosis (67–70). Many of these conditions accompany tumor progression, metastasis, and cancer treatments. In order for the tumor cells to survive and for the tumor to progress, proteins must be synthesized to cope with these stresses. Many of the mRNAs that encode for these proteins contain an IRES (Table 1), suggesting that IRES-mediated translation must play an important role in tumor progression and survival. Direct evidence supporting this model comes from several studies (15–17, 71). IRES-mediated translation was shown to promote cell survival and the formation of tumor emboli in inflammatory breast cancer (16, 17). Another study showed that treatment of a 3D ovarian cell culture with a PI3K/mTOR inhibitor (to globally downregulate cap-dependent protein synthesis) resulted in apoptosis of the majority of the cancer cells. Importantly, a sub-population of the cells that were resistant to treatment overexpressed survival proteins that contained IRESs (15). These data demonstrate that IRES-mediated translation may be required for cancer progression and survival.

Additional support for the role of IRES-driven translation in tumorigenesis comes from studies of a genetic disorder called X-linked dyskeratosis (X-DC). Patients with mutations in the *DKC1* gene, which causes X-DC, exhibit an increased susceptibility to cancer among other abnormalities (72). *DKC1* encodes for dyskerin, a pseudouridine synthase that isomerizes uridines on rRNA to pseudouridines in a sequence-specific manner. Biochemical evidence demonstrated that ribosomes with decreased pseudouridylation displayed a reduced affinity for IRES-containing mRNAs (73). Importantly, when rRNA pseudouridylation is reduced, there is a specific decrease in translation of some IRES-containing mRNAs: p27, XIAP, and Bcl-xL (74), while another IRES-containing mRNA, vascular endothelial growth factor (VEGF), is translationally induced (75). Reduced levels of p27, a tumor suppressor, could at least, in part, explain why patients with mutations in *DKC1* have an increased susceptibility to cancer (76). Altogether, these studies reveal a significant role for posttranscriptional control of gene expression during tumorigenesis that requires IRES-mediated initiation.

## Cap-Independent Translation in Apoptosis

### p53 IRES-Mediated Translation Is Required for p53 Induction of Cellular Senescence and Apoptosis

The p53 tumor suppressor protein is dysregulated in over half of all cancers. It is a transcription factor that controls the expression of protein coding genes as well as micro-RNAs (miRNAs) (77, 78). It plays a critical role in cellular responses to DNA damage and other stresses by inducing cell-cycle arrest and programed cell death (79). Failure to induce senescence or apoptosis following DNA damage results in genetic instability or inappropriate survival of damaged cells. Thus, inherited or spontaneous mutations in p53 contribute to tumorigenesis.

**Figure 1:**
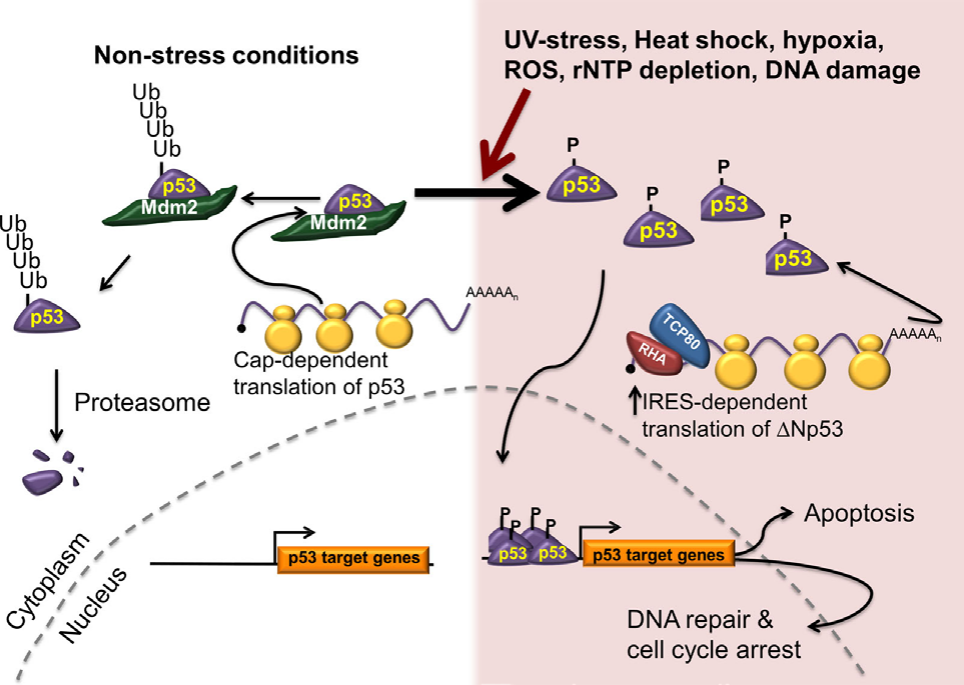
**IRES-mediated translation is required for cell survival and tumor progression**. Under normal conditions (left), p53 protein is synthesized predominately using a cap-dependent mechanism of initiation. The newly synthesized p53 protein is recognized by an E3 ubiquitin ligase (Mdm2), ubiquitinated, and degraded by the proteasome. In response to multiple stresses, p53 is phosphorylated, thus stabilizing it by preventing its interaction with Mdm2. In addition, IRES-mediated translation of the p53 mRNA is upregulated generating ΔNp53 and resulting in increased p53 levels. The phosphorylated p53 translocates to the nucleus to promote transcription of a number of genes involved in cell cycle arrest, DNA repair, and apoptosis.

Since p53 plays a vital role in controlling cellular functions, its activity is highly regulated. Optimal induction of growth arrest or apoptosis following DNA damage requires an increase in the intracellular p53 protein levels. Under normal growth conditions, the cell maintains a low level of p53 protein due to proteasomal targeting of p53 protein by the E3 ubiquitin ligase mouse double minute 2 (Mdm2) (80–82) (Figure 1, left). In addition, when Mdm2 and/or Mdm4 are bound to p53, they mask the transactivation domain. Following DNA damage or cell stress, the level of p53 protein in the cell increases, while mRNA levels remain constant (83). The increase in p53 protein level is controlled by two distinct mechanisms: stabilization of the p53 protein caused by the loss of Mdm2 recruitment and an increase in translation of the p53 mRNA (20, 84–86) (Figure 1, right). The *TP53* gene can express 12–13 different isoforms of the p53 protein with the major transcriptional start site generating a transcript with a 147 nucleotides long 5′UTR that contains an IRES (58, 87). IRES-mediated translation of the p53 mRNA generates ΔNp53 (also known as p54/47, p47, and Δ40p53) (25, 58, 88). Interestingly, IRES-mediated translation of p53 was shown to be important for increasing p53 protein levels following DNA damage in order to induce senescence (25, 26, 71).

The p53 IRES binds two IRES transacting factors, translational control protein 80 (TCP80), and RNA helicase (RHA) (25, 86), which were shown to modulate p53 IRES activity. If these proteins were either knocked down or overexpressed in cells, p53 proteins levels decreased or increased, respectively (25). This suggests that defective p53 regulation may be observed in cells with wild-type p53, but with dysregulated TCP80 or RHA. Thus, it is conceivable that many cancer cells that have wild-type p53 may still be unable to induce p53 due to defective p53 IRES-mediated translation (20, 25, 26).

### Apoptotic Protease-Activating Factor-1 Regulation Requires IRES-Mediated Translation during Apoptosis

Apaf-1 plays a central role in initiating the intrinsic or mitochondrial apoptotic pathway following cellular stresses, such as DNA damage (89–91). Under normal conditions, Apaf-1 is present in the cytoplasm as a monomer (92, 93). However, early apoptosis signals induce Apaf-1 oligomerization, which commits the cell to apoptosis. Briefly, exposure to proapoptotic stimuli triggers release of cytochrome *c* from the outer mitochondrial membrane into the cytoplasm. Binding of cytochrome *c* to dATP stimulates Apaf-1 oligomerization to form an apoptosome, which recruits and activates caspase 9 (91), which in turn induces the caspase cascade and commits the cell to apoptosis (91). Interestingly, Apaf-1 is expressed in all tissues except for muscle, which could be to avoid inappropriate activation of apoptosis when the mitochondria swell or degenerate following strenuous exercise (42, 94). Apaf-1 knockout mice are embryonic lethal due to reduced apoptosis resulting in an accumulation of neurons in the central nervous system causing many morphological abnormalities (95, 96). Overexpression of Apaf-1 increases the sensitivity of cells to proapoptotic stimuli (97). Cell lines that have Apaf-1 knocked out exhibit an increased propensity toward oncogenic transformation when c-Myc is overexpressed in cells (98). Significantly, a reduction in Apaf-1 expression is a negative prognostic marker for a variety of cancers including melanoma, cervical carcinoma, colon cancer, and acute myeloid leukemia (99, 100).

The Apaf-1 5′UTR is long, G-C rich, contains upstream start codons, has 56% homology between human and mouse, and was inhibitory to 40S scanning (42). This suggests that Apaf-1 is translationally regulated through a conserved mechanism. Indeed, the 5′UTR of Apaf-1 contains an IRES, and any possibility of cryptic promoter, cryptic splicing, or readthrough was ruled out (42). Furthermore, the Apaf-1 IRES was shown to be dependent on the ribosomal protein S25 (RPS25/eS25), which has been shown to be required for IRES-mediated translation, but has no affect on cap-dependent initiation (59, 101). Additionally, the Apaf-1 IRES is active if it is transfected into the cell as an RNA (102), which is highly significant since many cellular IRESs are only functional when they are transfected as a DNA. RNA transfection bypasses cellular splicing and transcription machinery, which are the major sources of false positives in the bicistronic reporter assay. Lastly, in response to ultraviolet C irradiation, an increase in Apaf-1 IRES activity correlates with an increase in Apaf-1 protein levels without a corresponding increase in mRNA levels (89), indicating Apaf-1 expression is translationally regulated. Furthermore, Apaf-1 protein levels are associated with increased sensitivity to ultraviolet-induced apoptosis (95, 103, 104).

Since proapoptotic stimuli triggers downregulation of cap-dependent translation (105, 106), IRES-mediated initiation of Apaf-1 ensures that Apaf-1 protein levels are maintained during apoptosis, which is necessary to ensure propagation of the caspase cascade (89, 107). Moreover, while many mRNAs that have cellular IRESs can be translated by both cap-dependent and cap-independent mechanisms, the 5′UTR of Apaf-1 is inhibitory for the scanning mechanism of initiation (42, 102). Therefore, the Apaf-1 mRNA represents an exquisite example of gene expression control in which mRNA is translationally repressed when cap-dependent translation predominates and is translationally upregulated upon stresses that downregulate cap-dependent translation such as apoptosis (108, 109). A possible mechanism for such regulation of Apaf-1 during apoptosis comes from studies of death-associated protein 5 (DAP5) protein. DAP5 is an eIF4G family member that gets activated, while other eIF4G proteins are inactivated during apoptosis by caspase cleavage (109, 110). Upon cleavage, DAP5 can stimulate IRES-mediated translation of Apaf-1 but not cap-dependent translation because it lacks the domain that binds eIF4E (the cap-binding protein).

Many cellular IRESs are cell type specific and Apaf-1 is no exception (111, 112). The Apaf-1 IRES activity is highest in neuronal cells (111). This cell type specificity is consistent with the fact that Apaf-1 protein is highly expressed in neurons, and the knockout mice exhibit defects in brain formation (95, 96). In fact, it was reported that the Apaf-1 IRES is significantly more active in neuronal cell lines compared to HeLa or HEK293T cells due to a stronger binding preference for the neuronal pyrimidine tract binding (nPTB) over the PTB1 that is expressed in non-neuronal cell types (111).

## IRES-Mediated Translation in Tumor Progression

### Cap-Independent Translation of c-Jun Is Required for Tumor Progression

The oncoprotein, c-Jun, is a component of the activator protein 1 (AP-1) transcription factor, which is involved in regulating proliferation, differentiation, growth, apoptosis, cell-migration, and transformation (113). AP-1 is regulated at many levels, which include dimer composition, transcriptional, translational, and posttranslational regulation (49, 114, 115). The c-Jun protein is known to stimulate transcription of components of the cell cycle, repress transcription of tumor suppressor genes such as *TP53*, and induce expression of metalloproteinases, which are proteolytic enzymes that promote growth, invasion, and metastasis of cancer cells (116). Surprisingly, c-Jun mRNA expression levels are only elevated in a few cancers (115, 117, 118). However, high c-Jun protein levels have been observed in glioblastoma, malignant melanoma, invasive breast cancer, and colorectal cancers without corresponding increases in mRNA levels or changes in protein stability. Likewise, the c-Jun protein levels are low in normal cells, but high in glioblastoma and melanoma cell lines (49, 115). Together, these data support a model whereby c-Jun expression is translationally controlled (49, 114, 115, 119).

The 5′UTR of c-Jun is 974 nucleotides long, conserved across species, and contains an IRES (49). Importantly, IRES-mediated translation of c-Jun mRNA resulted in accumulation of c-Jun protein without an increase in mRNA levels or changes in protein stability (120). IRES-mediated translation of c-Jun can be induced by loss of cell–cell contacts, such as when there is a loss of E-cadherin, which causes a disruption or restructuring of the cytoskeletal network (114, 120, 121). Disruption of the cytoskeletal network activates a signaling pathway that upregulates IRES-mediated translation of c-Jun and induces an invasive program (122, 123). Thus, IRES-mediated translation of c-Jun likely plays an important role in tumor progression, following the loss of adhesion molecules and/or restructuring of the cytoskeleton.

## Conclusion

Cap-dependent translation is the primary mechanism of initiating translation for the majority of mRNAs; however, cancer cells must adapt to many stresses that downregulate cap-dependent translation, including, but not limited to, hypoxia, nutrient deprivation, and DNA damage. In order for the cancer to progress, it must rely on other translational mechanisms, such as IRES-mediated translation, to support survival, growth, angiogenesis, and metastasis. Many genes that have been shown to be important in tumorigenesis have also had IRESs identified in their 5′UTR (Table 1). Thus, there is a need to better understand how IRES-mediated translation contributes to proteome changes in both healthy and tumor cells. This information will be critical for prognosis and development of more effective anticancer therapeutics.

## Author Contributions

SRT and BW contributed to the development, writing, and editing of the manuscript.

## Conflict of Interest Statement

The authors declare that the research was conducted in the absence of any commercial or financial relationships that could be construed as a potential conflict of interest.
